# Vitamin D deficiency linked to abnormal bone and lipid metabolism predicts high-risk multiple myeloma with poorer prognosis

**DOI:** 10.3389/fendo.2023.1157969

**Published:** 2023-04-27

**Authors:** Li Bao, Yu-tong Wang, Min-qiu Lu, Bin Chu, Lei Shi, Shan Gao, Li-juan Fang, Qiu-qing Xiang, Yue-hua Ding, Xi Liu, Xin Zhao, Meng-zhen Wang, Yuan Chen, Wei-kai Hu

**Affiliations:** Department of Hematology, Beijing JiShuiTan Hospital, The Fourth Clinical Medical College of Peking University, Beijing, China

**Keywords:** vitamin D, newly diagnosed multiple myeloma, cholesterol, ratio of vitamin D to β-CTX, poor survival

## Abstract

**Purpose:**

Vitamin D deficiency is frequent in patients with multiple myeloma (MM), however, its prognostic relevance in MM was rather inconclusive. We first investigated the association of vitamin D deficiency with abnormal bone and lipid metabolism in newly diagnosed multiple myeloma (NDMM), and next assessed the impact of serum ratio of vitamin D to carboxy-terminal telopeptide of type I collagen (β-CTX) on progression-free survival (PFS) and overall free survival (OS) in patients with NDMM.

**Methods:**

The data of 431 consecutive patients with NDMM at Beijing Jishuitan Hospital from September 2013 to December 2022 were collected and retrospectively reviewed through our electronic medical record system. The measurement of 25-hydroxyvitamin D in the blood is an indicator of an individual’s overall vitamin D status.

**Results:**

The serum levels of vitamin D were negatively correlated with β-CTX in NDMM patients. Of note, positive correlation between vitamin D and cholesterol levels in the serum was found in this study. The cohort (n = 431) was divided into two groups based on the serum ratio of vitamin D to β-CTX. Compared to the group with a higher vitamin D to β-CTX ratio, the group with a lower vitamin D to β-CTX ratio (n = 257, 60%) exhibited hypocholesterolemia, inferior PFS and OS, along with increased cases of ISS stage-III and R-ISS stage-III, a higher number of plasma cells in the bone marrow, and elevated serum calcium levels. Consistent with this, multivariate analysis confirmed that the vitamin D to β-CTX ratio was an independent unfavorable indicator for survival in NDMM patients.

**Conclusion:**

Our data demonstrated the ratio of vitamin D to β-CTX in the serum is a unique biomarker for NDMM patients to identify the high-risk cases with poor prognosis, which is superior to vitamin D itself for predicting PFS and OS in NDMM. Also, it is worth mentioning that our data on the connection between vitamin D deficiency and hypocholesterolemia might help clarify novel mechanistic aspects of myeloma development.

## Introduction

Vitamin D plays an important role in bone metabolism, lipid metabolism and cancer prevention (1–4). In clinical practice, the measurement of 25-hydroxyvitamin D in the blood is an indicator of an individual’s overall vitamin D status (5). Vitamin D regulates bone metabolism both through direct effects on osteoblasts and in an indirect manner through the control of calcium and phosphate homeostasis (4). Beyond bone health, 25-hydroxyvitamin D is used as a signaling molecule by almost every cell in the body (6). Recent clinical studies have revealed a consistent and strong association between vitamin D status and tumor progression and survival in several solid tumor (7). Vitamin D deficiency is extremely common in patients with multiple myeloma (MM), however, data on the association between vitamin D status and clinical outcomes of newly diagnosed multiple myeloma (NDMM) are rather inconsistent (8–12). There was a study showing vitamin D deficiency in NDMM is unrelated to survival or cytogenetics (12). Contrary to this, another clinical observation found that vitamin D deficiency corelated to poor overall survival in white NDMM patients, rather than African American patients, implying vitamin D status and its clinical relevance in MM are strongly influenced by racial differences (9, 12)**_。_** As vitamin D signaling has a key regulatory role in maintaining a healthy immune system, controlling cell proliferation, differentiation and growth, and inhibiting angiogenesis (8), keep clarifying the significance of vitamin D deficiency in myeloma development is undoubtedly needed.

Because NDMM is associated with bone metabolic dysfunction and abnormal lipid metabolism (13–16), this study hypothesizes that vitamin D status is related to particular bone metabolic dysfunction and abnormal lipid metabolism in patients with NDMM. Therefore, the aim of the current study was to explore the association between vitamin D deficiency and the changes of carboxy-terminal telopeptide of type I collagen (β-CTX) or cholesterol levels in the serum, as β-CTX and cholesterol respectively represent a surrogate for bone and lipid metabolism in clinical myeloma disease status, and vitamin D derived from cholesterol in the body (17). We therefore investigated whether the inversed relationship between serum vitamin D in NDMM patients could be observed. Furthermore, we aimed to assess the importance of vitamin D in MM by demonstrating serum ratio of vitamin D to β-CTX is an independent risk factor in NDMM.

## Patients and methods

### Study cohort

The data of 431 patients with newly diagnosed MM at Beijing Jishuitan Hospital (Beijing, P.R. China) from September 2013 to December 2022 were collected through our electronic medical record system and retrospectively reviewed. All patients had newly diagnosed MM without prior treatment. Patients with smoldering myeloma and those with non-plasma cell disorders were excluded. All of these research procedures were conducted in accordance with the Declaration of Helsinki and were approved by the local research ethics committee. Prior to data collection and analysis, the pan-informed consent form was written by each participant. The diagnosis criteria, disease staging, treatment regimens, and response evaluation of MM patients were performed according to the International Myeloma Working Group (IMWG) consensus. The first-line therapy for NDMM in this study consisted of Immunomodulatory drugs (IMiDs) and/or proteasome inhibitors, or others. The sum of complete response (CR), very good partial response (VGPR), and partial response (PR) was considered as the overall response (ORR). The other response included minor response (MR), stable disease (SD), and progression disease (PD). The eligible patients for autologous stem cell transplantation (ASCT) with PR and above response might undergo upfront ASCT post induction therapy. Laboratory data obtained at the time of diagnosis were extracted from the patient’s medical records. We collected data on clinical characteristics at diagnosis, height and weight, biochemical, endocrine, and bone metabolism findings, induction therapy regimen, and whether ASCT. These general and disease data were described in **Table 1**.

**Table 1:** Clinical characteristics at diagnosis.

Parameter	25-(OH)VD/β-CTX		*P* value
	≤25 (n=257)	>25 (n=174)	
Age, year	62 (27-85)	62 (33-88)	0.188
Sex, male/female	131/126	113/61	**0.005**
BMI, kg/m^2^	23.7 (16.0-32.5)	24.4 (15.9-34.4)	**0.004**
M protein types			0.958
IgG/IgA/IgD	107/69/9	79/42/7	
κ/λ/non-secretion	38/28/6	23/18/5	
ISS stage			<0.001
I/II/III/missed	62/60/126/9	78/51/37/8	
R-ISS stage			<0.001
I/II/III/missed	40/143/60/14	57/92/11/14	
Plasma cell in BM, %	32 (0-97)	17 (0-90)	**<0.001**
β2-microglobulin, mg/L	5.1 (1.2-63.7)	3.4 (0.7-38.5)	**<0.001**
LDH, IU/L	177 (66-1326)	167 (82-1121)	0.092
Ca, mmol/L	2.4 (1.6-4.5)	2.3 (1.7-3.6)	**<0.001**
P, mmol/L	1.29 (0.09-6.01)	1.17 (0.54-5.23)	0.283
ALP, IU/L	79 (19-372)	70 (20-605)	0.285
Albumin, g/L	37 (19-53)	40 (24-52)	**<0.001**
Cholesterol, mmol/L	3.6 (0.5-9.4)	4.2 (1-9.4)	**<0.001**
Triglyceride, mmol/L	1.3 (0.3-7.2)	1.5 (0.3-8.5)	**0.032**
HDL, mmol/L	1.0 (0.4-3.7)	1.1 (0.5-3.2)	0.279
LDL, mmol/L	2.1 (0.2-12.7)	2.5 (0.7-7.6)	0.761
First-line regimen			0.639
PIs based	141	88	
IMiDs based	34	14	
PIs+ IMiDs based	59	54	
Others	23	18	
ASCT	48	43	0.149

BMI, body mass index; BM, bone marrow; LDH, lactic dehydrogenase; ALP, alkaline phosphatase; HDL, high-density lipoprotein; LDL, low-density lipoprotein; ASCT, auto stem cell transplantation. The bold values presented P values ≤0.05

### Measurement of bone metabolism and lipid metabolism

The blood (serum) samples were adopted when the subject was fasting for over 8 hours and harvested on 7 AM. Bone turnover markers including P1NP (type 1 procollagen amino-terminal propeptide), β-CTX (bone resorption C-terminal telopeptides), type I collagen, osteocalcin, parathyroid hormone (PTH), 25-hydroxy vitamin D (25(OH) VD). The blood (serum) samples were measured for bone turnover markers by using electrochemiluminescence immunoassays (Roche Diagnostics GmbH, Sandhofer Strasse 116). Lipid metabolic markers included total cholesterol (TCH), triglyceride (TG), high-density lipoprotein cholesterol (HDL-C), and low-density lipoprotein cholesterol (LDL-C). When VD/β-CTX was chosen as an indicator to study the prognosis of MM, using its median value of 25 as the cut-off value.

### Follow-up and survival time

In this study, the patients with NDMM had complete follow-up and medical history data, unless death or withdrawal occurred with a median follow-up time of 36.9 months (95% CI: 32.3 to 41.5 months). First progression-free (PFS) time was defined as the time from diagnosis to relapse, death, or end of follow-up. Overall survival (OS) time was defined as the time from diagnosis of MM to death or end of follow-up. A retrospective comparative analysis of progression-free survival and overall survival between the low and high ratio of serum vitamin D to β-CTX in patients with NDMM was performed. All analyzes were made within the same clinical laboratory in our hospital.

### Statistical analyses

Statistical data were analyzed using the SPSS software (IBM Corp, Armonk, NY, USA, version 20.0). The primary endpoints of this study were PFS and OS, calculated by the Kaplan-Meier curve. The continuous variables and categorical clinical characteristics were compared using unpaired t-tests and Fisher’s exact test respectively. The affection of variables such as vitamin D levels, gender, diagnosis, age, and remission status on survival was assessed by multivariable Cox regression. P values less than 0.05 were considered significant.

## Results

### Reduced serum vitamin D level in patients with NDMM

**Figure 1:**
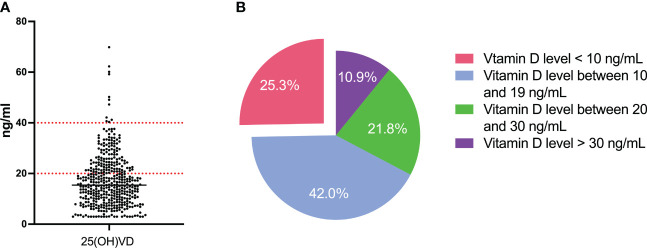
Level of vitamin D of NDMM patients. **(A)** The serum level of 25-OH-D3 of all patients; **(B)** Distribution of patients according to the serum level of 25-OH-D3.

Previous studies have shown that vitamin D status and its clinical relevance in MM are strongly influenced by racial differences (9, 18). Therefore, we first evaluated the serum level of vitamin D in Chinese patients with NDMM. There were 431 consecutive patients enrolled in the analysis. The median value of plasma Vitamin D level is 15.45 ng/ml, ranging from <3 ng/ml to 69.87 ng/ml. According to the Endocrine Society’s Clinical Practice Guideline (19), these patients were classified as follows: 47 (10.9%) patients were considered normal vitamin D with serum levels greater than 30 ng/mL, 94(21.8%)patients had vitamin D insufficiency (serum levels between 20–30 ng/mL), 181 (42%) patients had vitamin D deficiency (serum levels between 10–19 ng/mL) and 109 (25.3%) of patients had vitamin D severe deficiency (serum levels <10 ng/mL). These results are presented in **Figure 1**. Our data indicate that suboptimal serum levels of vitamin D (serum levels <30 ng/mL) were observed in the majority of the Chinese patients with NDMM (89.1%, 384 cases), which is consistent with previous reports on vitamin D deficiency in NDMM (8–10, 18, 20).

### The serum levels of vitamin D is positive correlated with cholesterol in NDMM patients

**Figure 2:**
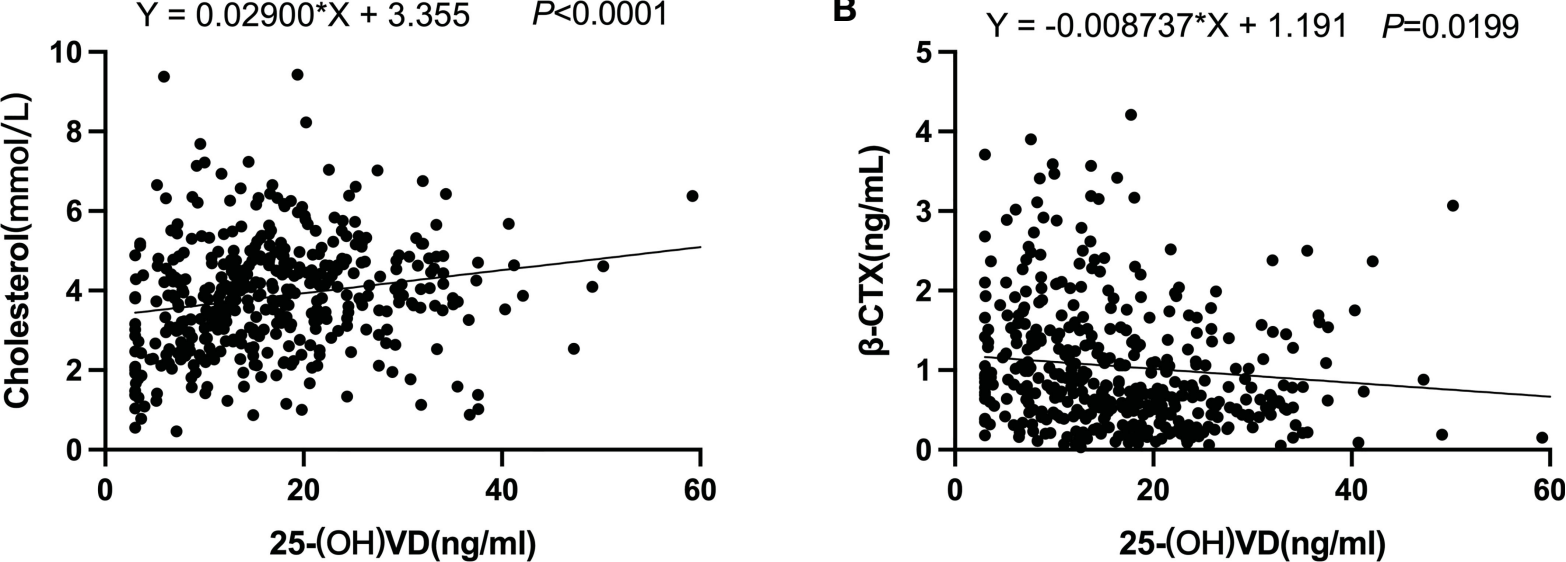
The correlation of vitamin D with cholesterol and β-CTX. **(A)** Cholesterol; **(B)** β-CTX.

Recently, the role of abnormal lipid metabolism in the development of myeloma has received increasing attention (13, 21–23). A nationwide population-based study has shown that patients with lower lipid levels and greater variability of HDL-C had an increased risk of developing MM (14). Cholesterol is one of several types of lipids that perform many important functions in the body: making cell membranes, many hormones, and vitamin D. Therefore, we investigated the correlation between serum levels of 25-OH vitamin D and cholesterol in patients with NDMM. As shown in **Figure 2A**, there was a striking correlation between 25-OH vitamin D and cholesterol. This finding provides new insight into clarifying the complex mechanisms involved in the development of myeloma.

### There is a negative correlation between vitamin D levels and β-CTX levels in the serum of NDMM patients

**Figure 3:**
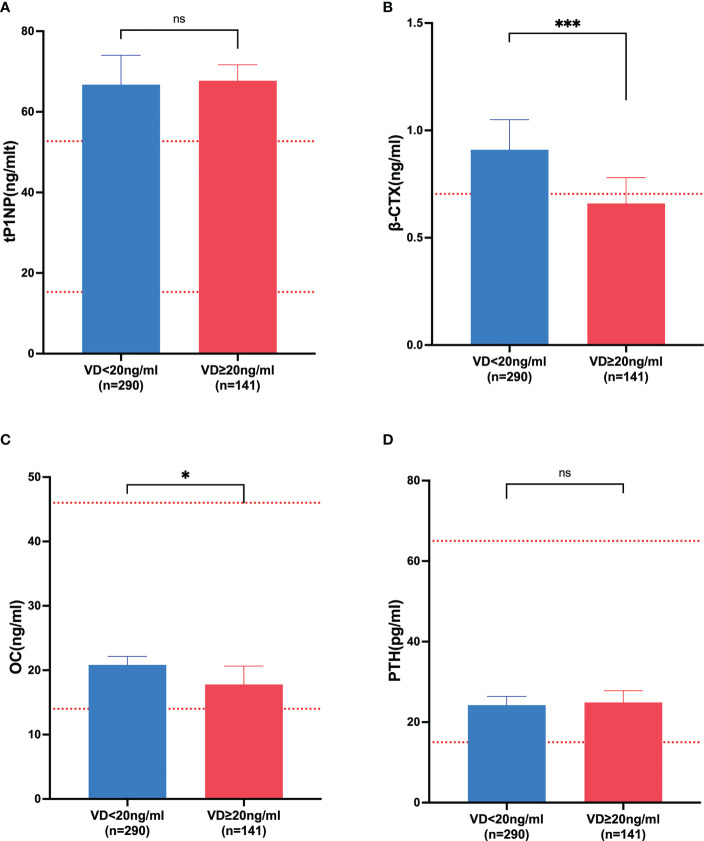
The markers of bone metabolism affected by vitamin D. **(A)** tP1NP; **(B)** β-CTX; **(C)** OC; **(D)** PTH. VD, vitamin D; tP1NP, type 1 procollagen amino-terminal propeptide; β-CTX, bone resorption C-terminal telopeptides; OC, osteocalcin; PTH, parathyroid hormone. * represented *p*-value ≤0.05; *** represented *p*-value < 0.0001; ns represented *p*-value >0.05.

Vitamin D, an important hormone for bone and immune health, is commonly deficient in MM patients. Because bone involvement is a central feature of MM and the serum levels of β-CTX represent a specific and sensitive marker capable of detecting the degree of bone resorption in myeloma, we next assessed the correlation between serum 25-OH vitamin D and β-CTX in patients with NDMM. The NDMM patient cohort (n = 431) was divided into two groups: 290 (67%) had 25-OH vitamin levels <20 ng/mL (low vitamin D status) and 141 (33%) had 25-OH vitamin levels ≥20 ng/mL (normal vitamin D status). As shown in **Figure 3**, serum levels of the β-CTX significantly (P <.05) increased in the group with low vitamin D status compared with the group with normal vitamin D status. Likewise, there is a negative correlation between vitamin D levels and β-CTX levels in the serum of NDMM patients (**Figure 2B**). These results suggested that vitamin D plays a critical role in bone metabolism with reducing activated bone resorption in NDMM.

### Decreased serum ratio of vitamin D to β-CTX in patients with NDMM is associated with an inferior PFS and OS

**Figure 4:**
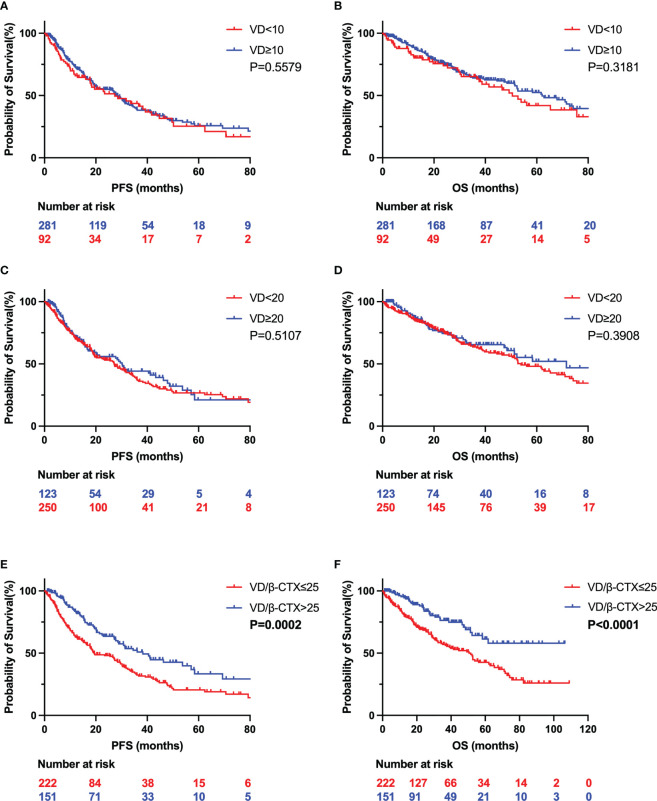
Kaplan Meier curves depicting outcomes of patients with values of Vitamin D and VD/β-CTX. The PFS **(A)** and OS **(B)** of the two groups divide by vitamin D levels of 10ng/ml. The PFS **(C)** and OS **(D)** of the two groups divide by vitamin D levels of 20ng/ml. The PFS **(E)** and OS **(F)** of the two groups divide by VD/β-CTX value of 25. VD, vitamin D; PFS, progression-free survival; OS, overall survival; β-CTX, bone resorption C-terminal telopeptides.

As mentioned above, the majority of myeloma patients are vitamin D deficient but its prognostic significance is inconclusive. Because the coexistence of low 25-OH vitamin D and high β-CTX levels and negative correlation between vitamin D levels and β-CTX levels in the serum of NDMM patients can be observed, we hypothesized that the serum ratio of vitamin D to β-CTX is superior on vitamin D itself for predicting PFS and OS in NDMM. As shown in **Table 1**, when the NDMM patients were divided into higher and lower vitamin D/β-CTX ratio groups, their vitamin D/β-CTX ratio was greater than 25 or not, with 59.6% of them in the lower VD/β-CTX value group and 40.4% in the higher VD/β-CTX value group. As expected, a lower serum ratio of vitamin D to β-CTX in patients with NDMM is associated with an inferior PFS and OS, when compared with the group with a higher ratio of vitamin D to β-CTX. In contrast, serum 25(OH)D levels were not associated with worsened overall survival in patients with NDMM. The detailed results are presented in **Figure 4**. These results indicate that the serum ratio of vitamin D to β-CTX, but not serum vitamin D levels, could act as a prognostic factor for patients with NDMM.

In the modern era of new medicine, new drugs in first-line regimens for NDMM significantly improved the PFS and OS of patients (24, 25). In the present study, there was no significant difference in first-line induction regimens between the two groups with high and low vitamin D/β-CTX ratios (**Table 1**), suggesting that the different prognosis(PFS and OS) of NDMM patients between the two groups with higher and lower vitamin D/β-CTX ratios was not related to the application of new drugs.

### Lower serum ratio of vitamin D to β-CTX exhibits high-risk clinical characteristics in NDMM

After observing serum ratio of vitamin D to β-CTX in patients with NDMM is associated with an inferior PFS and OS, we next analyzed the clinical data on clinical characteristics in NDMM to find the differences between higher and lower vitamin D/β-CTX ratio groups. As shown in **Table 1**, among NDMM patients, increased cases of ISS stage-III and R-ISS stage-III, higher plasma cell number in bone marrow, and elevated calcium in the serum were observed in lower VD/β-CTX group when compared to higher vitamin D/β-CTX ratio group. Consistently, lower lipid (cholesterol) levels in the serum of NDMM patients with lower vitamin D/β-CTX ratio group can be seen. The results demonstrate that serum ratio of vitamin D/β-CTX is a useful indicator for NDMM patients to identify the high-risk cases with cytogenetics.

### Univariate and multivariable analyses of prognostic factors for PFS and OS in NDMM patients

As serum ratio of vitamin D to β-CTX could be a prognostic factor for patients with NDMM, we further analyzed the risk factors for PFS and OS in patients with NDMM and performed univariate and multivariate logistic regression analyses on 431 NDMM patients. The detailed results are shown in **Table 2**. Univariate analysis suggested that Age≥65, Albumin ≤ 35, Renal dysfunction, Hypercalcemia, elevated LDH level, ISS III, R-ISS III, Gain 1q21, del 17p, MAF/IGH or MAFB/IGH, ASCT and VD/β-CTX at the time of MM diagnosis were risk factors for PFS in NDMM patients. The above factors were included in multivariate analysis, and the results confirmed that Age≥65, elevated LDH, Gain 1q21, del 17p, MAF/IGH or MAFB/IGH, ASCT and VD/β-CTX at the time of MM diagnosis were independent risk factors for PFS in NDMM patients. For OS in NDMM patients, univariate analysis suggested that Age≥65, Albumin ≤ 35, Hypercalcemia, elevated LDH level, ISS III, R-ISS III, Gain 1q21, del 17p, MAF/IGH or MAFB/IGH, ASCT and VD/β-CTX at the time of MM diagnosis were risk factors. The above factors were included in multivariate analysis, and the results confirmed that Age≥65, elevated LDH, ASCT and VD/β-CTX at the time of MM diagnosis were independent risk factors for OS in NDMM patients. These results indicate that decreased serum ratio of vitamin D to β-CTX in patients with NDMM was a poor prognostic factor for PFS (P = 0.024) and OS (P = 0.006).

**Table 2:** Cox regression analysis for PFS and OS.

Variants	PFS						OS					
	Univariate			Multivariate			Univariate			Multivariate		
	HR	95% CI	P value	HR	95% CI	P value	HR	95% CI	P value	HR	95% CI	P value
Sex	1.10	0.83-1.45	0.523				0.78	0.55-1.10	0.157			
Age≥65	1.60	1.20-2.12	**0.001**	1.44	1.03-2.03	**0.035**	2.14	1.51-3.05	**<0.001**	1.89	1.24-2.86	**0.003**
Albumin ≤ 35	1.35	1.01-.81	**0.042**	1.12	0.78-.61	0.531	1.32	0.92-1.89	0.135			
Renal dysfunction	1.74	1.20-2.52	**0.004**	1.21	0.74-1.98	0.461	1.92	1.24-2.99	**0.004**	0.99	0.56-1.76	0.971
Hypercalcemia	1.35	1.01-.82	**0.049**	1.12	0.77-.63	0.548	1.54	1.07-.20	**0.019**	1.33	0.86-2.05	0.200
LDH elevated	2.05	1.45-2.90	**<0.001**	1.81	1.23-2.66	**0.003**	2.5	1.68-3.78	**<0.001**	1.99	1.25-3.17	**0.004**
β-CTX>0.704	1.42	1.07-1.89	**0.016**	1.07	0.69-1.64	0.768	1.64	1.15-2.35	**0.007**	1.28	0.74-2.23	0.374
ISS III	1.83	1.38-2.43	**<0.001**	1.23	0.84-1.81	0.294	1.95	1.38-2.77	**<0.001**	1.10	0.68-1.76	0.699
R-ISS III	1.83	1.30-2.57	**0.001**	1.01	0.62-1.64	0.981	2.55	1.71-3.83	**<0.001**	1.53	0.87-2.70	0.143
CCND1/IGH	0.85	0.57-1.26	0.404				0.80	0.48-1.34	0.391			
Gain 1q21	1.67	1.24-2.28	**0.001**	1.69	1.21-2.36	**0.002**	1.60	1.10-2.33	**0.014**	1.23	0.81-1.88	0.336
del 17p	1.77	1.22-2.55	**0.002**	1.55	1.03-2.35	**0.038**	1.98	1.27-3.07	**0.002**	1.48	0.56-2.54	0.158
MAF/IGH or MAFB/IGH	2.54	1.19-5.42	**0.016**	2.53	1.13-5.67	**0.024**	3.31	1.34-8.18	**0.010**	1.92	0.73-5.07	0.187
FGFR3/IGH	1.28	0.82-1.99	0.286				1.14	0.63-2.08	0.666			
ASCT	0.41	0.26-0.60	**<0.001**	0.40	0.27-0.59	**<0.001**	0.29	0.17-0.51	**<0.001**	0.34	0.19-0.63	**0.001**
VD/β-CTX	0.58	0.43-0.78	**<0.001**	0.69	0.50-0.95	**0.024**	0.44	0.29-0.65	**<0.001**	0.54	0.34-0.84	**0.006**

PFS, progression free survival; OS, overall survival; ISS, international staging system; RISS, revised international staging system; LDH, lactate dehydrogenase; CI, confidence interval. The bold values presented P values ≤0.05

## Discussion

Vitamin D is an essential vitamin for everyone, ensuring the health of the immune system, bones and vital organs. But vitamin D deficiency is common in China, especially among the elderly. To this end, a consensus on the standardization of vitamin D fortified nutrient supplementation for the elderly and a consensus on the clinical application of vitamin D and its analogues have been formulated, and relevant publicity and education work has been carried out. These actions improve health policy by increasing knowledge about adequate vitamin D intake.

Although vitamin D levels varied widely by country, sex, and season, the associations between vitamin D levels and all-cause and cause-specific mortality were remarkably consistent. Evidence from observational studies suggests that circulating 25-hydroxyvitamin D is inversely associated with the risk of death from cardiovascular disease, cancer and other causes. Vitamin D has a greater impact on cancer mortality rates than on cancer incidence rates. Supplementation Vitamin D3 significantly reduces overall mortality in older adults (26, 27). A study also found that there is moderate evidence that vitamin D status is inversely associated with cancer mortality and death from respiratory diseases (28). High-dose chemotherapy (HDCT) with autologous stem cell transplantation (ASCT) is used in the consolidation of myeloma. A study showed that reduced serum vitamin D levels in myeloma patients who received HDCT/ASCT were associated with poor outcomes (29). With reference to the association between vitamin D status and cancer incidence rates, recent clinical study has revealed supplementation with vitamin D reduced the incidence of advanced (metastatic or fatal) cancer in the overall cohort, with the strongest risk reduction seen in individuals with normal weight (7). Vitamin D deficiency is extremely common in patients with MM. So far, the association between vitamin D status and the incidence of MM remains uncertain. Vitamin D measurement and supplementation are not standard care for myeloma patients, although known risk factors for vitamin D deficiency are common in these myeloma patients, including advanced age and insufficient sun exposure. Recently, the results of a multicenter, prospective, single-arm study showed that a vitamin D intervention, by using much higher doses of vitamin D3 than current guidelines recommend, resulted in a significant increase in vitamin D levels in patients with MM (30). In addition, peripheral neuropathy, a toxicity associated with antimyeloma therapy, appears to be attenuated by vitamin D3 supplementation (30). Notably, another *in vitro* study showed that vitamin D increases vulnerability to anti-CD38 antibodies by enhancing CD38 expression on myeloma cells (31). Therefore, the effectiveness of future vitamin D intervention strategies may have a beneficial role as adjuvant therapy in patients with MM, especially when combined with novel anti-myeloma agents.

The novelty of the current study lies in the observations that the serum ratio of vitamin D to β-CTX is a unique predictor of clinical outcome in patients with NDMM, which greatly helps to clarify the impact of vitamin D deficiency on clinical prognosis in NDMM patients. It has been demonstrated that vitamin D is deficient in the majority of myeloma patients. However, several studies’ findings for assessing the impact of vitamin D deficiency on clinical outcomes were controversial (8–12). The research comes from a study at the Mayo Clinic that showed that the deficiency of vitamin D was not related to worsened overall survival in patients with NDMM (10). Another group also supported this observation, showing vitamin D deficiency is unrelated to survival or high-risk cytogenetics (12). In sharp contrast to this, a clinical study, focusing on racial disparities between African American patients and white patients, observed that vitamin D deficiency is a predictor for poor overall survival in white patients but not African American with MM (9). To better elucidate the significance of vitamin D deficiency on the prognosis of myeloma, the serum ratio of vitamin D to β-CTX was successfully used in the current study for assessing myeloma prognosis. It is important to note that lower serum ratio of vitamin D to β-CTX not only predicts OS but also predicts PFS in patients with NDMM, which is also in line with the high-risk clinical characteristics and cytogenetics. The serum levels of β-CTX is sensitive to predicting subclinical bone disease before the onset of manifested lytic lesions, which is capable of detecting the degree of bone resorption in MM (32). Our findings highlight the importance of β-CTX levels in the relationship between vitamin D deficiency and clinical prognosis in patients with NDMM.

Another novel finding from our study is the strong connection between vitamin D deficiency and abnormal lipid metabolism in NDMM. Serum 25-OH vitamin D had a striking correlation with cholesterol can be observed. Likewise, the levels of cholesterol and triglycerides in the serum of NDMM patients were significantly lower in the group with lower ratio of vitamin D to β-CTX, when compared with the group with higher ratio of vitamin D to β-CTX. Over the last years, the important role of abnormal lipid metabolism in myeloma development has been recognized (14, 33). Recently, a nationwide population study of 3,527,776 subjects demonstrated that patients with lower lipid levels and greater variability in HDL-C were at increased risk of developing MM (14), our data support this conclusion. To our best knowledge, our report firstly revealed the association of bone metabolic dysfunction and abnormal lipid metabolism in myeloma. It has been demonstrated that deregulated metabolic pathways have implications for the immune cell function, tumor microenvironment, prognostic significance and drug resistance in MM (34). Our findings on the association of vitamin D and metabolic abnormalities in MM imply consequently inspire novel therapeutic interventions would be proposed for MM management.

Although this study demonstrated that vitamin D deficiency linked to abnormal bone and lipid metabolism predicts high-risk MM with a poorer prognosis, the following limitations are considered. Firstly, this was a single-center, retrospective, observational study, with data collected on 431 patients with NDMM over 9 years. Secondly, there is wide variability by race in the reported incidence of vitamin D deficiency. Furthermore, cultural or religious practices often lead to skin-covering clothing styles, further reducing the potential for skin vitamin D production, with a 5-fold increased risk of vitamin D deficiency (35). In the current study, all patients are Chinese people residing in northern China. Therefore, the question is whether such difficult to determine all incident occurring within the population of China or not, future international multicenter studies are needed to answer this important question.

In summary, our studies provide clinical evidence that demonstrates the serum ratio of vitamin D to β-CTX in patients with NDMM is a practical biomarker to recognize the high-risk cases with poor prognosis, and vitamin D deficiency is related to pathogenesis and progression in MM. Considering vitamin D could modulate the innate and adaptive immune responses through the vitamin D receptor expressed on immune cells (B cells, T cells, and antigen-presenting cells) (36), vitamin D may have clinical relevance as adjuvant therapy in the management of MM. Also, the finding of vitamin D linked to cholesterol provides a new direction for myeloma research.

## Data availability statement

The raw data supporting the conclusions of this article will be made available by the authors, without undue reservation.

## Ethics statement

The studies involving human participants were reviewed and approved by The ethics committee of Beijing Jishuitan hospital. The patients/participants provided their written informed consent to participate in this study.

## Author contributions

Conception and design: LB. Provision of study materials and patients: M-QL, LS, GS, L-JF, Q-QX. Collection and assembly of data: Y-TW, M-ZW, Y-HD, BC, W-KH, YC, XL. Data analysis and interpretation: Y-TW, LB. Manuscript writing: LB. Final approval and accountable for all aspects of the work: All authors. All authors contributed to the article and approved the submitted version.
